# High-titer anti-interferon-γ neutralizing autoantibodies linked to opportunistic infections in patients with adult-onset still's disease

**DOI:** 10.3389/fmed.2022.1097514

**Published:** 2023-01-09

**Authors:** Po-Ku Chen, Tsai-Ling Liao, Shih-Hsin Chang, Kai-Jieh Yeo, Chia-Hui Chou, Der-Yuan Chen

**Affiliations:** ^1^Rheumatology and Immunology Center, China Medical University Hospital, Taichung, Taiwan; ^2^College of Medicine, China Medical University, Taichung, Taiwan; ^3^Translational Medicine Laboratory, Rheumatology and Immunology Center, Taichung, Taiwan; ^4^Ph.D. Program in Translational Medicine and Rong Hsing Research Center for Translational Medicine, National Chung Hsing University, Taichung, Taiwan; ^5^Department of Medical Research, Taichung Veterans General Hospital, Taichung, Taiwan; ^6^Division of Infection, China Medical University Hospital, Taichung, Taiwan

**Keywords:** anti-interferon-γ autoantibodies, opportunistic infections, MCP-1, IFN-γ inducible protein-10 (IP-10), adult onset Still's disease (AOSD)

## Abstract

**Objective:**

Neutralizing anti-interferon (IFN)-γ autoantibodies are linked to opportunistic infections (OIs). To explore the association between anti-IFN-γ autoantibodies and OIs in patients with adult-onset Still's disease (AOSD), we aimed to examine the ability of these autoantibodies to blockade signal transducer and activator of transcription (STAT1)-phosphorylation and chemokines production.

**Methods:**

Serum titers of anti-IFN-γ autoantibodies were quantified using ELISA in 29 AOSD and 22 healthy controls (HC). The detectable autoantibodies were verified with immunoblotting assay, and their neutralizing capacity against IFN-γ-signaling was evaluated with flow-cytometry analysis and immunoblotting. IFN-γ-mediated production of supernatant chemokines, including monocyte chemoattractant protein-1 (MCP-1) and IFN-γ inducible protein-10 (IP-10), were measured by ELISA.

**Results:**

Among 29 AOSD patients, high titers of anti-IFN-γ neutralizing autoantibodies were detectable in two patients with OIs. Immunoblotting assay revealed more effective inhibition of STAT1-phosphorylation in THP-1 cells treated with sera from autoantibody-positive AOSD patients (56.7 ± 34.79%) compared with those from HC (104.3 ±29.51%), which was also demonstrated in flow-cytometry analysis (47.13 ± 40.99 vs. 97.92 ± 9.48%, *p* < 0.05). Depleted serum IgG from anti-IFN-γ autoAbs-positive AOSD patients with OIs restored phosphorylated STAT-1 upon IFN-γ treatment. Sera from autoantibody-positive AOSD patients more effectively inhibited IFN-γ-mediated production of MCP-1 (45.65 pg/ml) and IP-10 (22.44 pg/ml) than sera from HC (263.1 pg/ml and 104.0 pg/ml, both *p* < 0.05). Serum samples showing the strongest inhibition of IFN-γ-signaling were from two patients with high-titer autoantibodies and OIs.

**Conclusion:**

AOSD patients have a high positive rate and titers of anti-IFN-γ autoantibodies. The remarkable blockade effect of high-titer autoantibodies on IFN-γ-mediated STAT1-phosphorylation and chemokines could make these patients susceptible to OIs.

## Introduction

Interferon (IFN)-γ, a type II IFN (IFN-II) and pro-inflammatory cytokine produced by innate immune cells, is essential for the host's defense against infection with intracellular pathogens (1, 2). Although the biological mechanism behind autoantibodies formation against IFN-γ (anti-IFN-γ autoAbs) remains unclear, several studies have shown that these autoAbs have an inhibitory effect on IFN-γ signal transduction (3, 4). Accordingly, neutralizing anti-IFN-γ autoAbs are recognized as a cause of adult-onset immunodeficiency (AOID) and associated with increased risks of opportunistic infections (OIs) such as disseminated non-tuberculous mycobacteria (NTM), non-typhoid *Salmonella, Cryptococcus*, and v*aricella-zoster* virus (VZV), particularly in Asian populations (3–9). Hong et al. further revealed that anti-IFN-γ autoAbs titers were strongly associated with the severity of infections, which were likely related to the biological activity of anti-IFN-γ autoAbs (10).

Adult-onset Still's disease (AOSD), a systemic inflammatory disorder, is characterized by fever, rash, arthritis, variable organ involvement, and increased acute phase reactants (11–13). AOSD is also marked by elevated NLRP3-inflammasome-derived cytokines, including IL-1β and IL-18 (14), and Th1-derived cytokine such as IFN-γ (15, 16). IFN-γ-induced chemokines, including IFN-γ inducible protein-10 (IP-10, also known as CXCL10), may further amplify AOSD inflammatory responses and cutaneous manifestations (17). Besides, monocyte chemoattractant protein-1 (MCP-1), a C-C type chemokine with a pivotal role in host defense against pathogens by recruiting macrophages to the inflamed sites, is potentially involved in AOSD pathogenesis (18). Kawakami et al. also revealed that IL-12 and IL-18 synergistically induced IFN-γ production by the immune cells (19). Given that AOSD patients are at potential risk of opportunistic infections (20), it would be instrumental in examining the anti-IFN-γ autoAbs levels and their association with opportunistic infections in AOSD patients. However, the presence of anti-IFN-γ autoAbs or their clinical impact on OIs has yet to be explored in AOSD.

This pilot study aimed to use ELISA to assess the prevalence of anti-IFN-γ autoAbs in AOSD patients and verify their presence with the immunoblotting assay. We also investigated the blockade effects of anti-IFN-γ autoAbs on the IFN-γ signaling-mediated STAT1 transactivation and chemokines production. We finally examined the neutralizing ability of anti-IFN-γ autoAbs and their potential association with the occurrence of OIs in AOSD patients.

## Methods

### Patients and study design

In this prospective study, 29 Taiwanese patients who fulfilled the Yamaguchi criteria of AOSD (21) were enrolled consecutively. Systemic disease activity was assessed with a modified Pouchot score (22). This systemic activity score (range 0–12) assigns one point to each of 12 manifestations: fever, evanescent rash, sore throat, arthralgia or arthritis, myalgia, pleuritis, pericarditis, pneumonitis, lymphadenopathy, hepatomegaly or abnormal liver function, elevated leukocyte count ≧ 15,000/mm^3^, and serum ferritin levels >3,000 μg/L. The enrolled patients did not receive any IFN-γ treatment. Blood samples were obtained when the patients were at an active disease status, defined by the presence of two or more clinical manifestations or laboratory abnormalities; the timing was before the emergence of opportunistic infections. Twenty-two healthy volunteers who had no rheumatic disease were enrolled as healthy control (HC) subjects. This study was approved by the Institutional Review Board of Chinese Medicine University hospital (CMUH110-REC1-086), and informed consent was obtained from each participant according to the Declaration of Helsinki.

### Determination of serum titers of anti-IFN-γ autoAbs with ELISA

Each ten-milliliter whole blood sample was collected in a tube containing EDTA (BD Biosciences, San Jose, CA, USA) and centrifuged at 2,000 rpm for 10 min. According to the manufacturer's instructions, serum titers of IgG autoantibodies to IFN-γ were determined with ELISA (Cell Sciences, Newburyport, MA, USA). Referencing a previous report (23), we defined a “positive” ELISA result as an anti-IFN-γ autoAbs titer ≥48 U/ml and the cut-off value as the mean value plus 5-fold standard deviations (SDs) of HC subjects.

### Functional evaluation of anti-IFN-γ autoAbs with flow cytometry analysis

The blocking ability of anti-IFN-γ autoAbs was assessed with their effects on STAT1 phosphorylation on human monocytic cell lines (THP-1, BCRC 60430, the Bioresource Collection and Research Center, Taiwan). Serum samples (10%) from AOSD patients or HCs were firstly incubated with or without 10 ng/ml rhIFN-γ at 37 °C for 30 min. To examine the blocking ability of anti-IFN-γ autoAbs through IgG depletion, the serum samples (10%) from AOSD patients were depleted of IgG by using Pierce™ Albumin Serum Depletion Kits (Thermo Fisher Scientific, #89875). THP-1 cells were stimulated with the mixture according to previous reports with minor modifications (24, 25). These cells were washed twice with PBS and fixed with absolutely ice-cold methanol on ice for 10 min. After washing with 1%BSA, the cells were stained with phycoerythrin (PE) anti-human phospho-STAT1 (pY701) monoclonal antibody (clone A17012A, BD Biosciences San Diego CA, USA) for 30 min at room temperature. The population of PE-pSTAT1 was analyzed by flow-cytometer with FlowJo version 7.6 software.

### Functional evaluation of anti-IFN-γ autoAbs on THP-1 cells with Western blotting

Serum samples (10%) from AOSD patients or HCs were pre-incubated with or without 10 ng/ml rhIFN-γ at 37 °C for 30 min. THP-1 cells were stimulated with the mixture for 30 min, according to previous reports, with minor modifications (25, 26). After washing with PBS, the cell pellets were lysed with Cell lysis buffer (#9803, Cell Signaling Technology, Danvers, MA, USA) with Roche cOmplete™ protease inhibitor cocktail (Roche) and stored at −80°C until analysis. The cell lysates were separated using SDS-polyacrylamide gel electrophoresis and then transferred to a PDVF membrane. After blocking with 5% milk, immunoblots were performed by using specific anti-human phospho-STAT1 (pY701) monoclonal antibody (#9167), anti-human total-STAT-1 monoclonal antibody (#14994, Cell Signaling Technology, Danvers, MA, USA), and anti-human GAPDH antibody (Elabscience, Houston, TX, USA). The immunoblots were hybridized with HRP-conjugated goat anti-rabbit IgG (Jackson Immunology Research Inc, West Grove, PA, USA). The immunoreactive bands were visualized using an enhanced chemiluminescence detection system (Millipore, Billerica, MA, USA), and the band intensity was determined by Image J software.

### Determination of supernatant levels of MCP-1 and IP-10 derived from anti-IFN-γ autoAbs-treated THP-1 cells using ELISA

To examine an inhibitory activity of anti- IFN-γ autoAbs on the production of chemokines from IFN-γ treated THP-1 cells, serum samples (10%) from AOSD patients or HC were firstly treated with 10 ng/ml rhIFN-γ for 30 min and then co-cultured with THP-1 cells at 37°C for 24 h. The supernatants of cultured THP-1 cells were collected in 1.5 mL-Eppendroff tubes and centrifuged at 2,000 rpm for 10 min. The levels of MCP-1 (DY279, R&D Systems, Minneapolis, MN, USA) and IP-10 (DY266, R&D Systems, Minneapolis, MN, USA) were determined by the ELISA kit, according to the manufacturer's instructions.

### Determination of inflammatory parameters

Erythrocyte sedimentation rate (ESR) was determined using the Westergren method. Serum ferritin levels were determined using a chemiluminescent immunoassay sandwich method (two-site immunoenzymatic assay, Beckman Coulter, Inc., 250 S. Kraemer Blvd., Brea, CA 92821 U.S.A.), and C-reactive protein (CRP) levels using an immunoturbidimetric method (Beckman Coulter, Inc., 250 S. Kraemer Blvd., Brea, CA 92821 U.S.A.). Serum levels of IFN-γ and IL-18 were measured using magnetic multiplex particle-based assay (Multiplex MAP kits, EMD Millipore, Billerica, MA, USA) according to the manufacturer's instructions.

### Statistical analysis

The results were presented as the mean ± standard deviation (SD), the standard error of the mean (SEM), or the median (interquartile range). We performed the Pearson's χ^2^ test to examine the between-group difference of categorical variables. The Mann-Whitney U test, the Kruskal-Wallis test with a *post-hoc* Dunn's test, or One-Way ANOVA test using Bonferroni correction was used to compare different groups. The missing values were excluded from the statistical analysis. A two-sided probability of <0.05 was considered significant.

## Results

### Clinical characteristics of the enrolled participants

As illustrated in Table 1, there were no significant differences in the demographic data or body mass index between AOSD patients and HC participants. Two AOSD patients developed opportunistic infections: one with disseminated NTM (*Mycobacterium abscess*; lymphadenitis and pneumonia), non-typhoid *Salmonella* septicemia, and *Listeria monocytogenes* meningitis; and the other with disseminated NTM (*Mycobacterium avium-intracellulare* complex; lymphadenitis and pneumonia) and non-typhoid *Salmonella* septicemia. Both patients also had indeterminate results of QuantiFERON-TB In-tube (QFT-GIT), a commercialized *ex vivo* IFN-γ released assay.

**Table 1:** Demographic data, disease activity scores, and the used medications in AOSD patients and healthy control (HC) subjects^#^.

**Characteristics**	**AOSD** **(*n* = 29)**	**HC** **(*n* = 22)**
Age at study entry, years	49.0 ± 16.6	42.0 ± 11.7
Female, *n* (%)	25 (86.2%)	19 (86.4%)
Body mass index, kg/m^2^	22.7 ± 3.8	24.6 ± 7.0
Disease duration, years	4.6 ± 2.2	NA
AOSD activity score	4.28 ± 1.22	NA
The used medications at study entry		
Corticosteroids, mg/day	4.9 ± 3.3	NA
The used csDMARDs		
Methotrexate	12 (41.4%)	NA
Hydroxychloroquine	10 (34.5%)	NA
Cyclosporine	6 (20.7%)	NA
Azathioprine	2 (6.9%)	NA
The used biologics		
IL-6R inhibitor	3 (10.3%)	NA
Abatacept	1 (3.4%)	NA

# Data were expressed as mean ± SD or number (%). NA, not applicable.

AOSD, adult-onset Still's disease; csDMARDs, conventional synthetic disease-modifying anti-rheumatic drugs; IL-6R, Interleukin-6 receptor.

### Serum titers of anti-IFN-γ autoAbs and their association with serum IFN-γ levels or disease activity in AOSD patients

**Figure 1:**
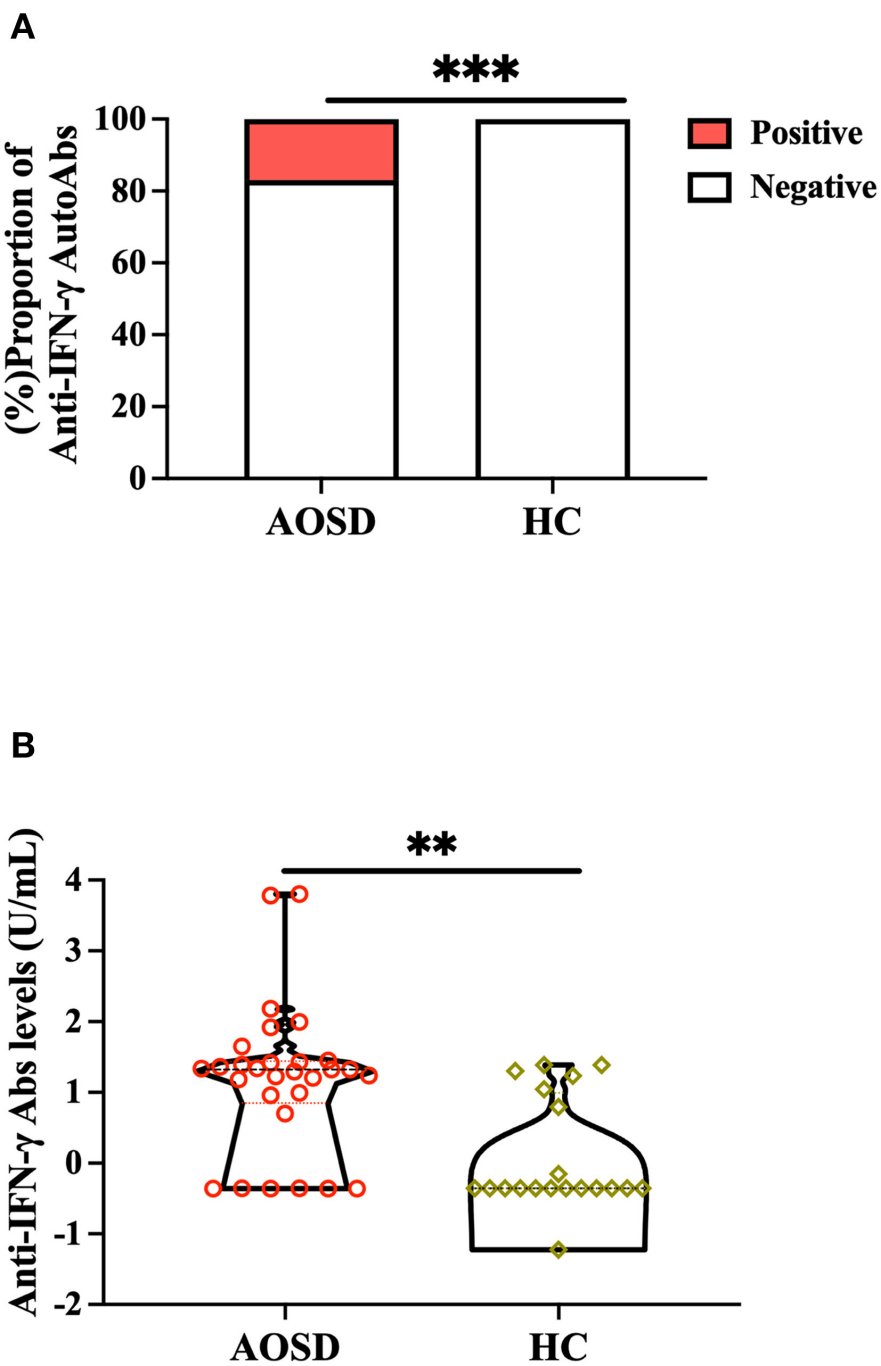
Comparison of **(A)** positive rates and **(B)** titers of anti-IFN-γ autoAbs between AOSD patients and HC participants. Anti-IFN-γ autoAbs was then determined by enzyme-linked immunosorbent assay (ELISA). ***p < 0.001, determined by the Chi-squared test **(A)**, or **p < 0.01, determined by the Mann-Whitney U test **(B)**. AOSD, adult-onset Still's disease; HC, healthy control; IFN-γ, interferon-γ.

Significantly higher proportions of AOSD patients (5/29, 17.2%) had positive anti-IFN-γ autoAbs compared with HC (0/20, 0.0%, *p* < 0.001, Figure 1A). Significantly higher titers of anti-IFN-γ autoAbs were also observed in AOSD patients [median 20.97 U/ml, interquartile range (IQR) 7.01–27.56 U/ml) than HC (0.44 U/ml, IQR: 0.44–9.87 U/ml, *p* < 0.001, Figure 1B). There was a trend of lower IFN-γ levels observed in anti-IFN-γ autoAbs-positive patients compared with autoAbs-negative patients (mean, 0.47 vs. 58.8pg/mL, *p* = 0.216, Supplementary Table 1). AOSD patients with anti-IFN-γ autoAbs had significantly higher disease activity (the modified Pouchot score) and inflammatory parameters, including ESR, CRP, ferritin levels, and IL-18 levels, than those without autoAbs (Supplementary Table 1). Besides, a significantly higher dose of corticosteroids was prescribed in autoantibody-positive patients than in autoantibody-negative patients. However, there was no significant difference in the use of conventional synthetic disease-modifying anti-rheumatic drugs (csDMARDs) or biologic DMARDs between AOSD patients with and without anti-IFN-γ autoAbs.

### The inhibitory effects of neutralizing anti-IFN-γ autoAbs on IFN-γ-mediated STAT activation

**Figure 2:**
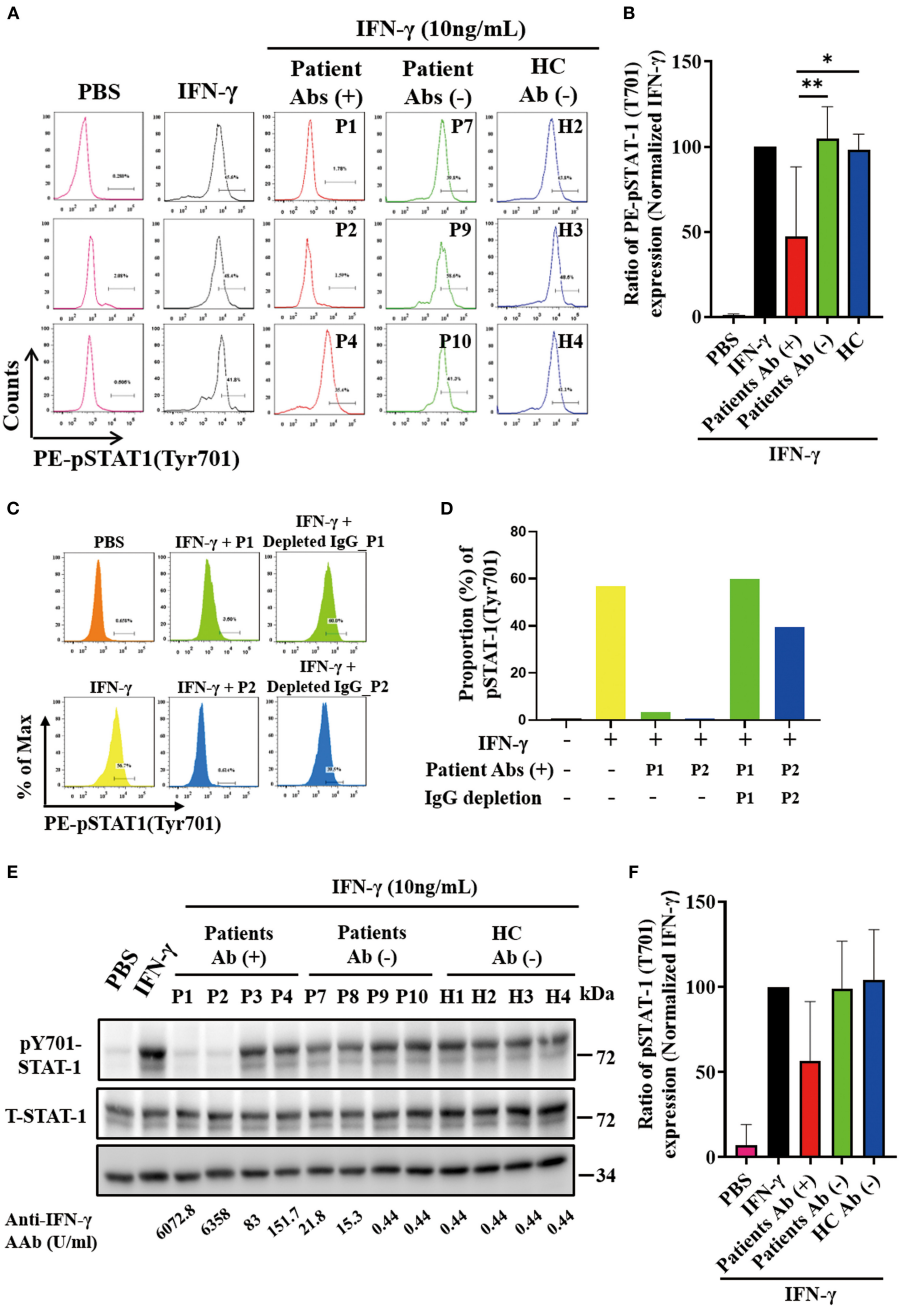
Effective neutralization of IFN-γ signaling by anti-IFN-γ autoAbs. **(A)** The representative histograms of the inhibitory effects on the IFN-γ-induced PE (phycoerythrin)-STAT1 phosphorylation in THP-1 cells treated with serum samples from autoAbs-positive AOSD patients (P1, P2, and P4), autoAbs-negative patients (P7, P9, and P10), or HC subjects (H2, H3, and H4). **(B)** Comparison of pSTAT1 intensity after inhibition with serum samples from three groups subjects. **(C, D)** IgG depletion of serum (10%) from autoAbs-positive AOSD patients (P1 and P2) were restored the IFN-γ-induced STAT1 phosphorylation on THP-1 cells. **(E)** The inhibitory effects on IFN-γ-induced STAT1 phosphorylation on THP-1 cells treated with serum samples from autoAbs-positive AOSD patients (P1-P4), autoAbs-negative patients (P7-P10), or HC subjects (H1-H4). **(F)** Comparison of phosphorylated STAT1 (pSTAT1) intensity after inhibition with serum from three groups subjects. Bars and error bars indicate the mean and standard error of mean, respectively. The p-values were determined by One-Way ANOVA test using Bonferroni correction. *p < 0.05, **p < 0.01.

As shown in the flow cytometry analysis, serum samples from autoAbs-positive AOSD patients could more effectively suppress the STAT1 phosphorylation (47.13 ± 40.99%) compared to those of HC (97.92 ± 9.48%, *p* < 0.05) (Figures 2A, B). The serum samples with the strongest inhibition effect on the STAT1 phosphorylation were from two AOSD patients (P1 and P2) who had high-titer anti-IFN-γ autoAbs and opportunistic infections (1.72 and 3.29%, respectively). Reflecting the neutralizing ability of IgG autoantibodies, depletion of IgG from the sera of anti-IFN-γ autoAbs-positive AOSD patients (P1 and P2) resulted in the restoration of phosphorylated STAT-1 upon IFN-γ treatment (Figures 2C, D). Besides, serum samples from AOSD patients P3-P5, who had low-titer autoAbs but no OIs, showed a greater capacity to block STAT1 phosphorylation (76.9 ± 6.36%) compared with those from the autoAbs-negative AOSD group (104.9 ± 18.63%, *p* = 0.0518).

In the immunoblotting assay, serum samples from autoAbs-positive AOSD patients could also more effectively suppress the STAT1 phosphorylation on THP-1 cells (56.70±34.79%), particularly the samples from two patients (P1 and P2) with high-titer anti-IFN-γ autoAbs (22.61 and 16.91%, respectively), compared to samples from HC (104.3 ± 29.51%, *p* = 0.057) (Figures 2E, F).

### Inhibitory effect of anti-IFN-γ autoAbs on IFN-γ-mediated production of chemokines (MCP-1 and IP-10)

**Figure 3:**
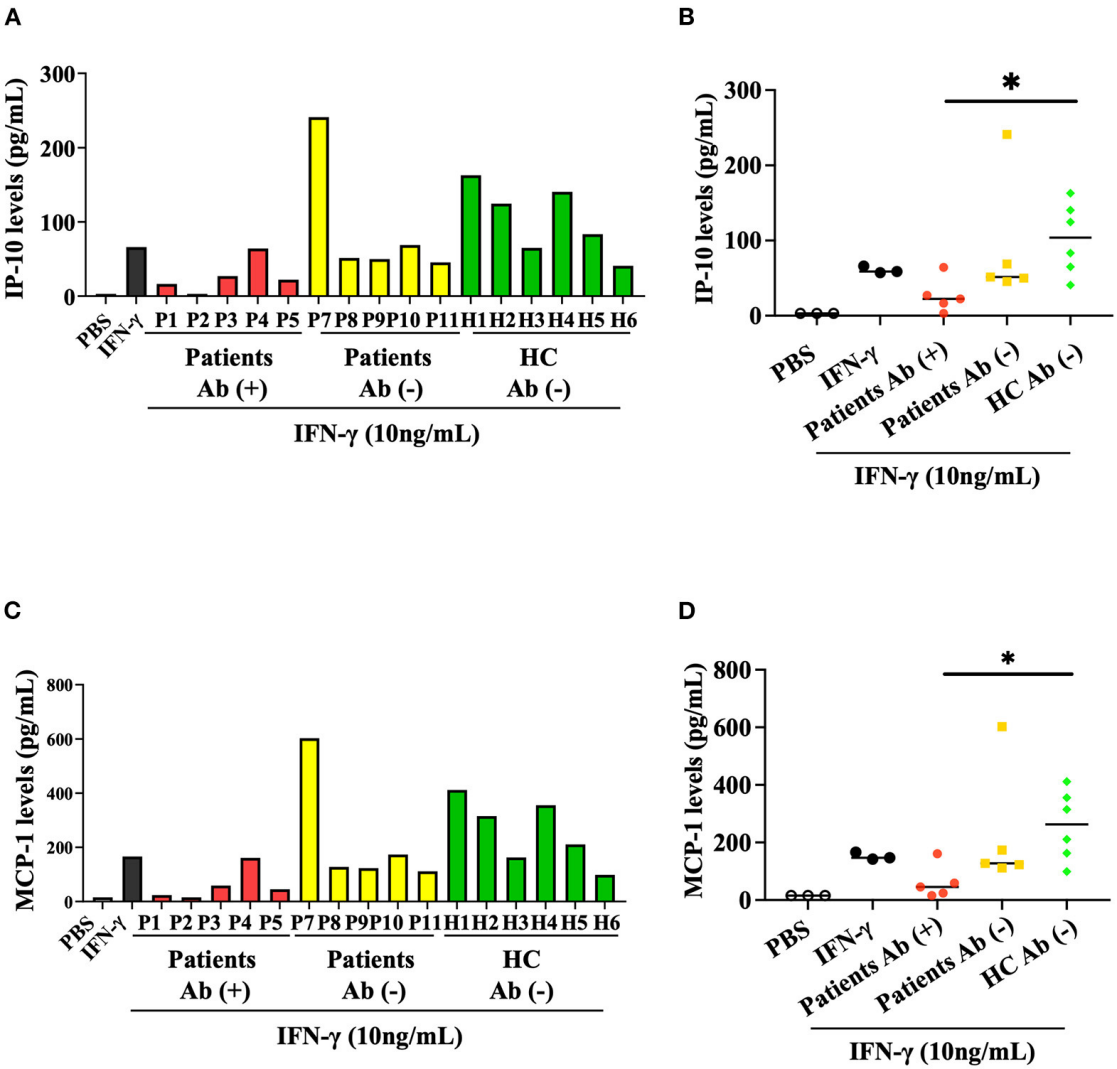
Inhibitory effect of anti-IFN-γ autoAbs on IFN-γ-mediated chemokines. **(A)** The inhibitory effects of anti-IFN-γ autoAbs on IFN-γ-mediated IP-10 production in THP-1 cells treated with serum samples from autoAbs-positive AOSD patients (P1-P5), autoAbs-negative patients (P7-P11), or HC subjects (H1-H6). **(B)** Comparison of the supernatant levels of IP-10 on THP-1 cells treated with serum samples from three groups subjects. **(C)** The inhibitory effects of anti-IFN-γ autoAbs on IFN-γ-mediated MCP-1 production in THP-1 cells treated with serum samples from three group subjects. **(D)** Comparison of the supernatant levels of IP-10 on THP-1 cells treated with serum samples from three groups subjects. Data are presented as box-plot diagrams, with the box encompassing the 25^th^ percentile (lower bar) to the 75^th^ percentile (upper bar). The horizontal line within the box indicates median value for each group. *p < 0.05, vs. HC, determined by the Kruskal-Wallis test using a *post-hoc* Dunn's test. IP-10, IFN-γ inducible protein-10; MCP-1, monocyte chemoattractant protein-1.

We then evaluated the inhibitory effect of anti-IFN-γ autoAbs on the IFN-γ-mediated production of chemokines, including IP-10 and MCP-1. Our results showed that serum samples from autoAbs-positive AOSD patients could more significantly inhibit IFN-γ-mediated production of IP-10 (median 24.79 pg/ml, IQR: 13.30–38.25 pg/ml) than samples from HC (104.0 pg/ml, IQR: 59.03–146.0 pg/ml, *p* < 0.05, Figures 3A, B). Similarly, MCP-1 production was more significantly inhibited by serum samples from autoAbs-positive AOSD patients (median, 52.38 pg/ml, IQR: 22.09–89.70 pg/ml) compared to those from HC (263.1pg/ml, IQR: 147.0–369.5 pg/ml, *p* < 0.05, Figures 3C, D). The serum samples showing the strongest inhibitory effects on the production of the chemokines were from two AOSD patients (P1 and P2) who had high-titer anti-IFN-γ autoAbs and OIs (IP-10: 16.69 and 3.13 pg/ml; MCP-1: 24.24 and 15.63 pg/ml, respectively).

## Discussion

Growing evidence suggests a close link between neutralizing anti-IFN-γ autoAbs and OIs (3–10, 27), yet their relationship in AOSD has not been explored. Herein, we are the first to demonstrate the significantly higher prevalence and titers of anti-IFN-γ autoAbs in AOSD patients compared with HC participants. The serum samples from high-titer anti-IFN-γ autoAbs-positive AOSD patients could effectively block the STAT1-phosphorylation and suppress the IFN-γ-mediated production of MCP-1 and IP-10. Besides, the most conspicuous inhibition of IFN-γ signaling was observed in the sera of two AOSD patients with both high-titer anti-IFN-γ autoAbs and OIs. These findings suggest that high-titer anti-IFN-γ autoAbs are biologically functional and may contribute to OIs in AOSD patients.

Although the mechanisms for the emergence of autoantibodies in AOSD remain unknown (28), anti-IFN-γ autoAbs were detectable in serum samples from 17.2% of AOSD patients. It has been proposed that endogenous anti-cytokine antibodies may be part of an immune regulatory response to hyper-inflammation or due to abundant cytokine exposure (28, 29). Resonated with this hypothesis, we revealed significantly higher disease activity and serum IL-18 levels in anti-IFN-γ autoAbs-positive patients compared with anti-IFN-γ autoAbs-negative patients. Since the anti-IFN-γ autoAbs could neutralize IFN-γ, our autoAbs-positive patients had lower IFN-γ levels (mean, 0.47 pg/mL) than autoAbs-negative patients (58.8 pg/mL). The non-significant difference is probably due to the small sample size. Besides, emerging evidence supports that anti-cytokine antibodies can modulate disease activity in inflammatory diseases (30). Similar to previous findings that anti-IFN-γ autoAbs were associated with increased disease activity and interferon signature in patients with systemic lupus erythematosus (31), the presence of anti-IFN-γ autoAbs in AOSD patients was associated with higher disease activity. Notably, this association was observed in a small cohort of AOSD patients, which needed further validation.

We then performed flow cytometry analysis to validate the biological function of anti-IFN-γ autoAbs in AOSD patients and revealed the neutralizing capacity of these autoAbs through the blockade of STAT1 phosphorylation. Similar downregulation of STAT1 protein expression was observed in THP-1 cells treated with sera from high-titer autoAbs-positive AOSD patients in the immunoblotting assay. Moreover, the serum samples producing the strongest inhibition of the STAT1 phosphorylation were from two AOSD patients who had high-titer anti-IFN-γ autoAbs and opportunistic infections. In comparison, the sera from our AOSD patients with low-titer anti-IFN-γ autoAbs showed a limited capacity for inhibiting STAT1 phosphorylation; thus, opportunistic infections were not observed in these patients. Our results were consistent with previous studies which reported a functional blockade of IFN-γ-mediated antimicrobial immunity by the high-titer anti-IFN-γ autoAbs (3, 32). These findings indicate that the high-titer anti-IFN-γ autoAbs may contribute to infections through functional neutralization of the IFN-γ-mediated signaling.

After STAT1 phosphorylation, IFN-γ could activate the transcription of genes of cytokines or chemokines that play a crucial role in antimicrobial activity. Patel et al. demonstrated that high-titer anti-IFN-γ autoAbs could effectively suppress IFN-γ-mediated downstream production of cytokines, including tumor necrosis factor (TNF)-α and IL-12, in East Asian women with disseminated NTM (3). Krisnawati et al. also revealed that treatment with NTM patients' sera significantly blocked the IFN-γ-induced production of TNF-α, IFN-γ, MCP-1, and IP-10 (26, 32). Given that the anti-IFN-γ autoAbs titers were inversely correlated with IP-10 and MCP-1 levels in our AOSD patients, we also assessed the effects of anti-IFN-γ autoAbs on the IFN-γ-mediated production of these chemokines. Resonated with the findings of Krisnawati et al. (26), the sera from our high-titer autoAbs-positive AOSD patients could more effectively inhibit the IFN-γ-mediated production of MCP-1 and IP-10 than those from HC. Furthermore, the serum samples that showed the strongest inhibitory effect on the production of chemokines were from two patients with high-titer anti-IFN-γ autoAbs and OIs. The indeterminate results of the QFT-GIT assay in these two patients also support the findings that anti-IFN-γ autoAbs might reduce the released IFN-γ levels in the QFT-GIT assay (33). MCP-1 could contribute to antimycobacterial inflammatory response by attracting monocytes and T lymphocytes (34), and Palucci et al. has revealed the inhibitory effect of IP-10 on mycobacterial growth (35). Gathering the evidence from other studies (3, 26, 32, 34, 35) and ours, high-titer anti-IFN-γ autoAbs may reduce antimicrobial activity at least partly through counteracting the IFN-γ-mediated production of cytokines/chemokines.

Despite the novel findings, there are some limitations of this study. The sample size of AOSD was small, an inherent limitation of this rare disease (36). We were unable to identify the absolute anti-IFN-γ autoAbs titers for predicting the occurrence of OIs, probably due to an insufficient number of patients with these infections. Given the higher dose of corticosteroids prescribed in our autoAbs-positive patients than in autoAbs-negative patients, the titer and neutralizing capacity of anti-IFN-γ autoAbs might be influenced by the therapeutic agents and the clinical characteristics when the blood samples were collected. Finally, the OIs may result from the immunosuppressive effects of the used medications or inherited mutations of IFN-γ-signaling-related genes, which were not evaluated in our AOSD patients. Therefore, a future large-scale study with sufficient statistical power is needed to validate this finding and its clinical implementation. Besides, anti-IFN-γ autoAbs-associated AOID was prevalent in the Asian population (6, 7), so our findings will need to be further validated in ethnically matched control populations.

In conclusion, we are the first to reveal that anti-IFN-γ autoAbs were detectable in 17.2% of AOSD patients, and high-titer autoAbs were associated with OIs through their blockade effects on IFN-γ-mediated STAT1-phosphorylation and chemokines. Our results would add AOSD to the list of diseases with the presence of neutralizing anti-IFN-γ autoAbs. It is clinically significant to beware that AOSD patients with increased neutralizing anti-IFN-γ autoAbs are at increased risk of OIs. Early detection of anti-IFN-γ autoAbs might help guide therapeutic interventions. Cyclophosphamide or rituximab therapy has been reported to reduce anti-IFN-γ autoAbs titers effectively; therefore, they might be an alternative treatment for autoAbs-positive patients with a refractory opportunistic infection, such as disseminated NTM infection (37–40).

## Data availability statement

The raw data supporting the conclusions of this article will be made available by the authors, without undue reservation.

## Ethics statement

The Institutional Review Board of our hospital approved this study (CMUH110-REC1-086). The patients/participants provided their written informed consent to participate in this study.

## Author contributions

P-KC conceived and designed the study, acquired the laboratory data, performed the data analysis, and drafted the manuscript. T-LL conducted the experiments, acquired the laboratory data, and performed data analysis. S-HC, K-JY, and C-HC acquired the clinical data and performed the data analysis. D-YC conceived and designed the study, acquired the clinical data, performed data analysis, and revised the manuscript. All authors have read and approved the final manuscript.
