# Supplementary material for: High-titer anti-interferon-γ neutralizing autoantibodies linked to opportunistic infections in patients with adult-onset still's disease
Source: Front Med (Lausanne). 2023 Jan 9;9:1097514. doi: 10.3389/fmed.2022.1097514 (PMC9868624; doi:10.3389/fmed.2022.1097514)
Supplement: Supplementary file 1 [file Table_1.docx]

**Supplementary Table 1.** Demographic data, disease activity scores, and the used medications in AOSD patients with and without anti-IFN-γ autoantibodies.

| **Characteristics** | Anti-IFN-γ autoAbs **(+)**  **(n=5)** | Anti-IFN-γ autoAbs **(-) (n=24)** |
| --- | --- | --- |
| Age at study entry, years | 44.6 ± 14.7 | 50.0 ± 17.1 |
| Female, n (%) | 5 (100%) | 21 (87.5%) |
| Systemic activity score | 6.0 ± 0.7*** | 3.9 ± 1.0 |
| ESR, mm/1^st^ hour | 69.2 ± 17.7*** | 23.0 ± 18.5 |
| C-reactive protein, mg/dL | 9.1 ± 3.2 ** | 2.2 ± 4.3 |
| Ferritin levels, ng/mL | 2647 ± 2208** | 467 ± 566 |
| Interleukin-18 levels, pg/mL | 4001 ± 3198* | 1193 ± 1931 |
| IFN-γ levels, pg/mL | 0.47 ± 0.19 | 58.84 ± 209.8 |
| The used medications at study entry |  |  |
| Corticosteroids, mg/day | 10.0 ± 3.1*** | 4.1 ± 2.6 |
| The used csDMARDs |  |  |
| Methotrexate | 4 (80.0%) | 8 (33.3%) |
| Hydroxychloroquine | 2 (40.0%) | 8 (33.3%) |
| Cyclosporine | 1 (20.0%) | 5 (20.8%) |
| Azathioprine | 0 (0.0%) | 2 (8.3%) |
| The used biologics |  |  |
| IL-6R inhibitor | 2 (40.0%) | 1 (4.2%) |
| Abatacept | 0 (0.0%) | 1 (3.4%) |

**^#^**Data were expressed as mean ± standard deviation or number (%).

AOSD: adult-onset Still’s disease; IFN: interferon; autoAbs: autoantibodies; ESR: erythrocyte sedimentation rate; csDMARDs: conventional synthetic disease-modifying anti-rheumatic drugs; IL-6R: Interleukin-6 receptor

^*^p<0.05, ^**^p<0.01, ^***^p<0.001, vs. anti-IFN-γ autoAbs **(-)** group, as determined by Mann-Whitney U test.
